# Holding cancer in line: the role of the electron transport chain in tumor-associated macrophages

**DOI:** 10.3389/fimmu.2026.1802495

**Published:** 2026-03-16

**Authors:** Alessia Zotta

**Affiliations:** 1 Laboratory of Tumor Inflammation and Angiogenesis, Center for Cancer Biology, Department of Oncology, Katholieke Universiteit Leuven, Leuven, Belgium; 2 Laboratory of Tumor Inflammation and Angiogenesis, Center for Cancer Biology, Vlaams Instituut voor Biotechnologie, Leuven, Belgium

**Keywords:** cancer immunology, electron transport chain (ETC), immunometabolism, mitochondria, tumor associated macrophage (TAM)

## Abstract

Tumor-associated macrophages (TAMs) are a highly heterogeneous population of innate immune cells that is widely enriched in the tumor microenvironment (TME). By suppressing anti-cancer immunity, TAMs sustain tumor growth, metastasis development and contribute to therapy resistance. Due to their remarkable plasticity, TAMs can be reprogrammed towards immune-stimulatory phenotypes, representing a compelling therapeutic option. The mitochondrial electron transport chain (ETC) is central in fueling macrophage metabolism by coupling electron flow with proton transfer to produce Adenosine Triphosphate (ATP). During inflammation, remodeling of the ETC has been shown to regulate macrophage polarization and cytokine production. However, how ETC perturbations influence macrophage phenotypes in other diseases, as during cancer progression and within a nutrient-restricted environment remains largely unexplored. In this mini-review, we examine the role of the ETC and its individual respiratory complexes in governing tumor-associated macrophage behavior, their involvement in tumor immunity, and we discuss the potential to exploit this axis for innovative immunotherapeutic strategies, while also considering current challenges and limitations.

## Introduction

As key players of the innate immune system, macrophages rapidly respond to danger signals through phagocytosis and secretion of cytokines and chemokines that participate to the recruitment and differentiation of other immune cell types (1). Many works have shown that macrophages are characterized by a huge heterogeneity, from their ontology to their activation state, which underlies their functional diversity (2, 3). Within the cancer microenvironment, tumor-associated macrophages represent a predominant immune population (4). Conditioned by the external milieu, TAMs foster tumor progression, therapeutic resistance (5), and metastatic dissemination via extracellular matrix (ECM) remodelling, angiogenesis, immunosuppression, and promotion of cell migration (6). These malignant features define them as prognostic factors and promising targets for immunotherapy (7).

**Figure 1:**
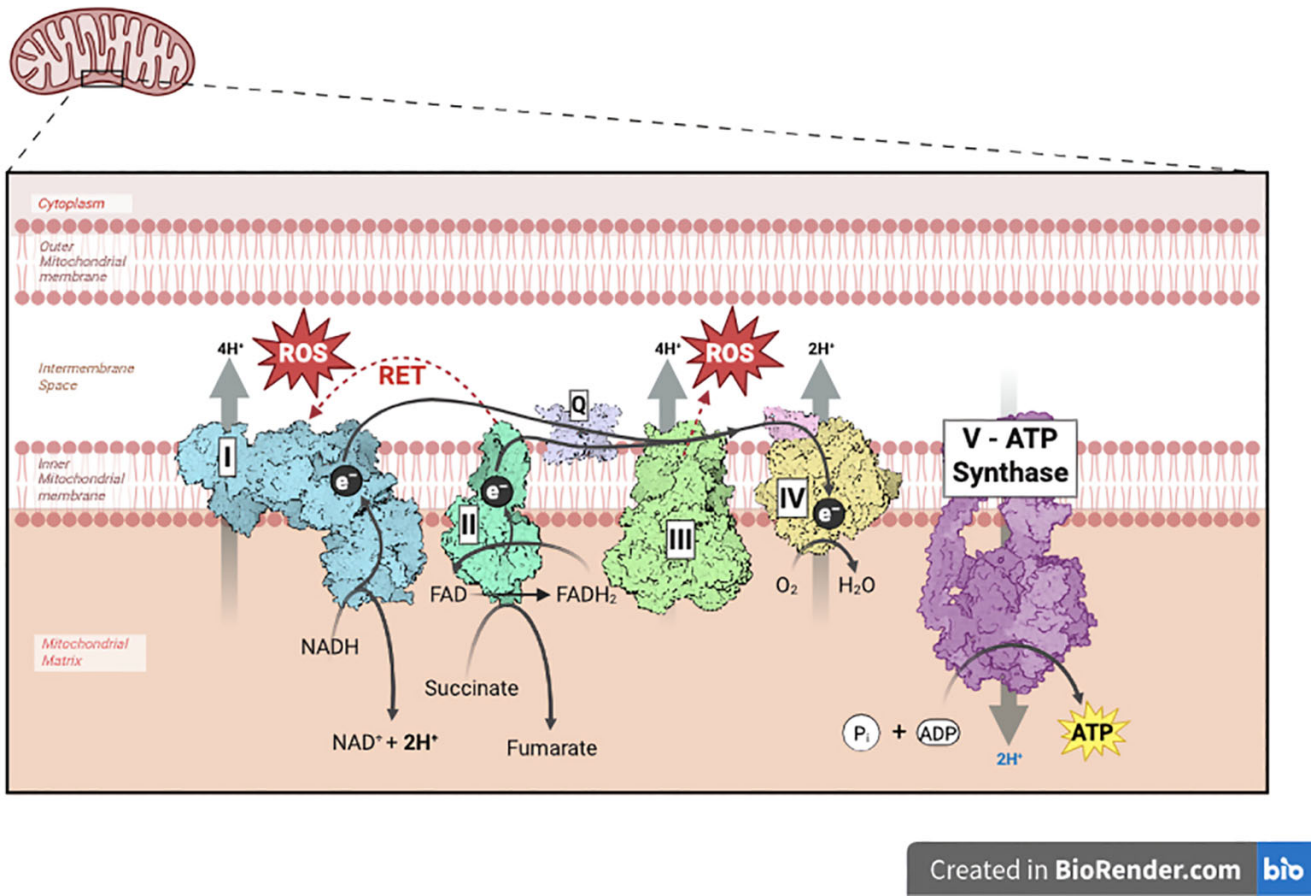
Overview of the mitochondrial electron transport chain in TAMs. NADH is oxidized and donates electrons to Complex I, which in turn reduces coenzyme Q. During this process, four protons are pumped from the mitochondrial matrix into the intermembrane space. The reduced CoQ then transfers electrons to Complex III, which subsequently reduces cytochrome c while also pumping four protons across the membrane. Cytochrome c carries the electrons to Complex IV, where oxygen is reduced. Finally, the accumulated protons in the intermembrane space flow back into the matrix through Complex V, driving the synthesis of ATP from ADP and inorganic phosphate. Notably, Complex I (via reverse electron transport) and Complex III are responsible for approximately 75% of superoxide and hydrogen peroxide production in mammalian mitochondria. NADH, nicotinamide adenine dinucleotide; CoQ, coenzyme Q; O_2_, oxygen; ATP, adenosine triphosphate; ADP, adenosine diphosphate; P_i_, inorganic phosphate; RET, reverse electron transport; O_2·_^-^, superoxide anion; H_2_O_2_, hydrogen peroxide; ROS, reactive oxygen species; e^-^, electron; FAD/FADH_2_, flavin adenine dinucleotide/1,5-dihydro-flavin adenine dinucleotide; H^+^, proton.

Oxidative phosphorylation (OXPHOS) in mitochondria is fuelled by the electron transport chain (8), situated in the inner mitochondrial membrane and formed by five multi-subunit Complexes (I-V), which pump protons from the mitochondrial matrix to the intermembrane space (IMS), generating ATP (**Figure 1**). Complex I (NADH coenzyme Q reductase) and Complex II (succinate dehydrogenase; SDH) transfer electrons from Nicotinamide Adenine Dinucleotide, reduced form (NADH) and succinate respectively to ubiquinone (CoQ) and reduce it to ubiquinol (CoQH2) (8). CoQH2 freely diffuses within the membrane and donates electrons to Complex III (cytochrome bc1 complex), which transfers them through cytochrome c (Cyt c) to Complex IV (cytochrome c oxidase), where oxygen (O_2_) is reduced to water (H_2_O). Complexes I, III and IV produce the protonmotive force that is used by the FoF1 ATP synthase (Complex V) to synthesize ATP (9). During this process, reactive oxygen species (ROS) are produced from Complex I (as electron leakage under reverse electron transport, RET) and Complex III. As ROS prematurely reacting with oxygen, superoxide (O_2_•−) and H_2_O_2_ (10) are generated and can act as signalling intermediates in cellular adaptation but can also promote oxidative stress.

This mini review examines the contribution of the ETC and its individual complexes to TAMs biology, synthesizes recent findings on how mitochondrial metabolism shapes TAMs function, and discuss strategies to therapeutically reprogram them towards anti-tumoral phenotypes. Elucidating TAMs immunometabolism may reveal novel avenues for cancer immunotherapy.

## Complex I

TAMs resemble anti-inflammatory macrophages, as they express canonical markers (i.e. Cluster of Differentiation (CD) 206, CD301, Arginase 1 (Arg1), Programmed Death Ligand (PD-L)1) (11). In addition to this, it is well documented that pro-tumoral TAMs produce cytokines and factors that promote immunosuppression, such as Interleukin (IL)-10, IL-35, and Transforming growth factor (TGF)-β (7). Due to the current lack of studies examining TAMs directly *in vivo* or following isolation from tumors, most available data derive from *in vitro* models using macrophages stimulated with IL-4, IL-13, or IL-10, or cultured in presence of tumor-conditioned media (TCM) of various immortalised cancer cell lines.

In IL-4-polarised Bone marrow-derived macrophages (BMDMs), pharmacological inhibition of Complex I activity by rotenone boosted the expression of *Arg1* and Chitinase-like 3 gene (*Ym1*), while failing to suppress the transcription of Mannose receptor C-type 1 (*Mrc1*, also called CD206), and *Retnla* (Resistin-like molecule, also called RELM alpha) (12). In another study, rotenone was able to restrain IL-10 and Monocyte Chemoattractant Protein (MCP)-1 production from macrophages in the same activation state (13).

Metformin, clinically available as a treatment for Type 2 diabetes (14), has been identified as a Complex I inhibitor (15). Pre-treatment of human monocyte-derived macrophages (MDMs) with Metformin before IL-4 stimulation did not affect the polarization towards an anti-inflammatory phenotype and decreased IL-10 and C-C motif Chemokine Ligand (CCL)-17 production (16).

Overall, evidence from polarization models (e.g., IL-4/IL-13/IL-10 or tumor-conditioned media) suggests that Complex I may regulate effector functions as production of specific cytokines and chemokines, possibly through secondary mechanisms. Although this has been demonstrated *in vitro*, it remains unclear whether similar effects are recapitulated within the tumor microenvironment.

## Complex II

Recent insights about Complex II-dependent regulation of anti-inflammatory macrophages come from a study from Gobelli et al. indicating a role for Complex II in the regulation of IL-10 production (17). The failure in IL-10 secretion in Lipopolysaccharide (LPS)-primed *Sdha-/- and Sdhb-/-* BMDMs was accompanied by a decrease in phosphorylation of Signal transducer and activator of transcription (STAT) 3 on the Tyrosine 705 residue. Treatment with dimethyl malonate (DMM), inhibitor of SDH, was equally altering the anti-inflammatory cytokine (17). Given the well-established heterogeneity of macrophages, we cannot exclude that such mechanisms observed in LPS-stimulated macrophages *in vitro*, may also characterize specific TAM subsets *in vivo* under certain pathological conditions.

In another study, DMM has been shown to increase pro-tumoral markers, like *Arg1, Mrc1* and *Ym1* in IL-4 alone and IL-13 alone stimulated BMDMs, through a STAT6-dependent mechanism. Together with this, DMM caused an impairment in the production of the chemokines Cxcl1 and Cxcl5, and the *Fizz1* marker. The enhanced effect of the compound was confirmed with the analysis of the immune profile of peritoneal exudate macrophages (18) from wild type (WT) mice which were injected intraperitoneally with DMM.

Similarly to Complex I, there are currently no studies directly demonstrating the effect of SDH inhibition on TAMs or its consequences for tumor growth control. Because SDH does not directly participate in the proton pumping process, it is important to clarify the impact of its inhibition in immune cells. Notably, succinate—the substrate of Complex II—has been widely described as a cancer cell–derived metabolite (19), together with lactate and citrate, suggesting that metabolic crosstalk between malignant cells and TAMs may influence macrophage function, in cancer cell conditioned media (20). Succinate has been shown to polarise peritoneal macrophages towards TAMs through the binding to its receptor Succinate receptor 1 (SUCNR1) (19) and, in another study, succinate-loaded tumor-cell derived microparticles have been demonstrated to rewire pro-tumoral TAMs towards and anti-tumoral phenotype (21). Considering these conflicting findings, it will be interesting to understand which is the effect of tumor cell-derived succinate on the ETC in TAMs, succinate being the optimal substrate for Complex II. Considering these conflicting findings, further studies are needed to determine the impact of tumor cell–derived succinate on the electron transport chain in TAMs, given that succinate serves as the primary substrate for Complex II.

Itaconate, a Tricarboxylic Acid Cycle (TCA)-derived metabolite by decarboxylation of cis-aconitate, has been documented as an immunomodulatory metabolite with anti-inflammatory and anti-microbial properties (22). One of its targets has been identified with SDH, as itaconate possess structural similarity with succinate. Blocking of SDH by itaconate prevents the oxidation of succinate to fumarate, stopping the generation of complex I–driven mitochondrial reactive oxygen species (mitoROS) (23). Itaconate has been extensively studied in cancer immunology, yet its role remains controversial, as it has been reported to both promote and suppress tumor growth through effects on myeloid-derived suppressor cells (MDSCs), tumor cells, and macrophages (24–27). However, whether it regulates the immunosuppressive functions of TAMs by impairing their bioenergetic machinery remains unexplored.

Briefly, Complex II emerges as a regulator of macrophage anti-inflammatory functions, particularly IL-10 production via STAT3 signalling, as its genetic or pharmacological inhibition reduces IL-10 and alters macrophage activation. At the same time, SDH inhibition can enhance pro-tumoral polarization markers (Arg1, Mrc1, Ym1) through STAT6 while suppressing certain chemokines, highlighting context-dependent effects. Importantly, metabolic cues from tumors—especially succinate and itaconate—can shape macrophage behaviour via both receptor signalling (SUCNR1) and potential effects on Complex II, though conflicting evidence suggests both pro- and anti-tumoral outcomes. Collectively, these findings depict an intricate role for Complex II in TAMs biology, with significant gaps in *in vivo* validation.

## Complex III

In a study from last year, the inhibition of Complex III by Suppressor of site III_QO_ Electron Leak (S3QEL) 1.2, a specific suppressor of Complex III-derived ROS (28), was reported to reduce the presence of IL-10^+^ macrophages in B16F10 tumor-bearing mice. Simultaneously, the same treatment increased Major Histocompatibility Complex (MHC) II expression in these cells, suggesting a reprogramming of macrophages towards immune activation in the absence of Complex III activity. The use of S3QEL 1.2 *in vivo* improved survival of tumor-bearing animals and decreased endpoint tumor size compared to the control group (28). Despite the encouraging outcome, off-target effects of S3QEL 1.2 in cancer cells cannot be excluded. Mechanistically, *in vitro* experiments showed that S3QEL 1.2 pre-treatment impaired the presence of nuclear Activator Protein (AP)-1 transcription factor and subsequently IL-10 transcription and release by LPS-activated BMDMs.

The findings of this study have been supported by the work of Stoolman et al., including the TLR3 and TLR4 stimulation of BMDMs derived from QPC-KO (LysM^Cre/Cre^; QPC^Fl/Fl^; Rosa26^Ai14/Ai14^ i.e. mice with floxed alleles of the mitochondria CIII subunit *Uqcrq* and with TdTomato reporter) mice (29). Upon LPS and poly(I:C) engagement, QPC-KO BMDMs showed decreased IL-10 release compared to BMDMs derived from QPC-HET controls. Indeed, restoration of respiration in QPC-KO mice by crossing them with *C. intestinalis* AOX (ROSA26^SNAPf-AOX^) mice, rescued also IL-10 release in after macrophage activation. Moreover, to gain further understanding of Complex III involvement in pro-tumoral response of macrophages, the researchers performed RNA sequencing (RNA-seq) analysis of QPC-HET and QPC-KO BMDMs 18 hours post–IL-4 stimulation. In this analysis, IL-4 driven reprogramming appeared unaffected by Complex III deficiency, as *Arg-1*, *RELM-α, PD-L2*, CD301 and CD206 were not different between QPC-HET and QPC-KO BMDMs (29).

The present studies are unique in the field, opening new avenues to investigate the role of Complex III in the immune compartment of tumors. Complex III–derived mitochondrial ROS play a key role in regulating macrophage immunosuppressive functions, particularly IL-10 production and MHC II expression, and shift macrophages towards a more immunostimulatory phenotype, improving tumor control *in vivo*. Genetic disruption of Complex III similarly decreases IL-10 production, which can be rescued by restoring mitochondrial respiration. Notably, despite this effect on cytokine production, Complex III does not appear essential for IL-4–driven macrophage polarization, indicating that it selectively regulates immunosuppressive outputs rather than overall macrophage identity.

## Complex IV

Within the crowd of macrophages infiltrating the tumor mass, there are existing conserved TAMs subpopulations which can either stop or promote tumor establishment. One of these is represented by Interferon (IFN)-TAMs, expressing many Interferon-stimulated genes, MHC II and linked to immunostimulatory functions (30). IFN-TAMs have been shown to correlate with better response to Immune Checkpoint Blockade (ICB) (31). Clark et al. recently demonstrated that melanoma-derived INF-TAMs have high amount of NDUFA4L3, a paralog of one of the core subunits of Complex IV, and of the miRNA miR-147, both coded by the same transcript. This result was thereafter confirmed by IFN stimulation of BMDMs *in vitro*. The group discovered that miR-147 and NDUFA4L3 worked together to silence the subunit NDUFA4 of Complex IV, leading to inactivity of the complex. Mice with *Ndufa4* depletion in the macrophage lineage (through the use of LysM-Cre mice) were characterized by decreased melanoma and colorectal carcinoma tumor growth rates and increased frequencies of intratumoral Natural Killer (NK) and CD8+ T cells, together with an enhanced production of Granzyme B and IFN-γ. To understand the mechanism behind this effect, *Ndufa4*-deficient BMDMs treated with IFNγ were analyzed and presented lower membrane potential, mitoROS and membrane mass, consistent with mitochondrial dysfunction. Indeed, subsequent release of mtDNA was responsible for the triggering and amplification of the cyclic GMP-AMP synthase (cGAS)-Stimulator of Interferon Genes (STING) pathway. The administration of synthetic miR-147 in combination with anti-Programmed Death (PD)-1 antibody in animals bearing B16F10 tumors impaired tumor growth, thus leveraging the importance of targeting the ETC in IFN-TAMs to improve ICB potency.

This study provides strong evidence that mitochondrial complexes intrinsically regulate the immune functions of TAMs. Here, IFN-TAMs exhibit a distinct metabolic program involving suppression of mitochondrial Complex IV activity via coordinated action of miR-147 and NDUFA4L3, leading to inhibition of the NDUFA4 subunit. This mitochondrial dysfunction promotes mtDNA release and activation of the cGAS–STING pathway, enhancing anti-tumor immunity. Despite the unique bioenergetic features of IFN-TAMs in melanoma, strategies to selectively target this subset without impacting other immune cell types remain to be developed.

## Complex V

IL-4-polarised macrophages and TAMs have been reported to highly rely on oxidative phosphorylation (32). Oligomycin covalently binds to the subunit c of the of the F_o_ portion of Complex V (33) and it is used as an inhibitor for the entire respiratory machinery. One of the few studies available to date reported that oligomycin pre-treatment of LPS-stimulated BMDMs reduced the production of IL-6, IL-1β, MCP-1, and IL-10, likely due to effects on the electron transport chain and cell viability. In contrast, IL-4–dependent cytokine responses were not significantly affected (13), a finding that was confirmed in another independent study (12). The link between Complex V impairment and a diminished cytokine response might indicate that energy depletion is a barrier for cellular functioning and immune response in macrophages.

While blocking Complex V broadly reduces cytokine production in LPS-stimulated macrophages—likely due to impaired energy supply and cell viability—it has minimal impact on IL-4–driven polarization and dependent responses. Overall, these findings suggest that mitochondrial ATP generation is critical for sustaining inflammatory outputs, whereas anti-inflammatory macrophage identity is less reliant on Complex V activity.

## Discussion

**Figure 2:**
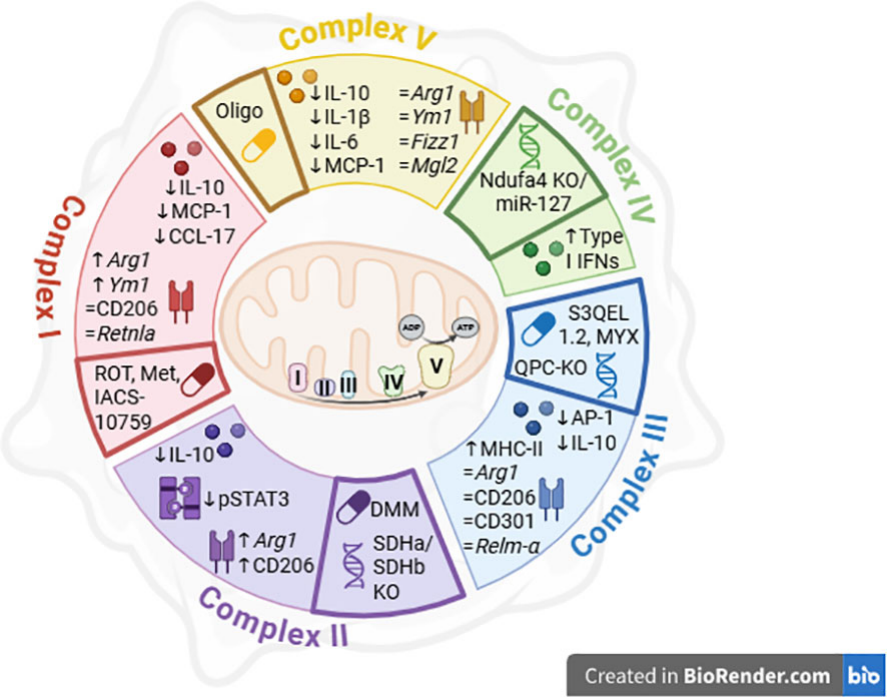
Functionalities of TAMs are regulated by mitochondrial respiratory complexes. The inhibition of Complex I by Rotenone increases the expression of *Retnla, Ym1*, without affecting the expression of the surface marker CD206 and *Retnla*, and decrease IL-10 and MCP-1 generation in IL-4 stimulated macrophages. In the same activating conditions, Metformin impairs IL-10 and CCL-17 production. Moreover, if Complex II is blocked by dimethyl malonate or in SDHa or SDHb knock out conditions, it hinders phosphorylation of STAT3 and IL-10 while it boosts the factors CD206 and *Arg1* in LPS-treated BMDMs. IL-10 generation is also limited by Complex III impairment with S3QEL 1.2 *in vitro* and *in vivo*, together with an elevation in MHC II levels. Macrophages derived from QPC-KO mice and stimulated with TLR4 and TLR3 ligands showed the same impairment in IL-10 release. In IL-4 treated QPC-KO macrophages, the classical anti-inflammatory markers are not affected. Furthermore, when the subunit Ndufa4 of Complex IV is suppressed by miR-127, the production of type I IFN is promoted. Finally, Complex V suppression by oligomycin limits IL-1β, IL-6, MCP-1 and IL-10 after TLR4 engagement by LPS but does not affect the IL-4-dependent response. Pharmacological and genetical approaches for ETC targeting are represented in bold boxes. IL, Interleukin; MCP-1, Monocyte chemoattractant protein 1; CD, cluster of differentiation; ROT, rotenone; Met, Metformin; Arg1, Arginase 1; Ym1, Chitinase 3-like 3; Retnla, Relm-α, Fizz1, Resistin-like α/RELM-α; STAT, Signal transducer and activator of transcription; Mrc1, Mannose receptor C-type 1/CD206; DMM, Dimethyl malonate; SDH, Succinate dehydrogenase; AP-1, Activator protein 1; MHC, Major histocompatibility complex; MYX, Myxothiazol; S3QEL 1.2, Suppressor of site III_QO_ Electron Leak; QPC, UQCRQ, Ubiquinol-cytochrome c reductase, complex III subunit VII; miR, miRNA; IFN, Interferon; Ndufa4, NADH dehydrogenase ubiquinone 1 α subcomplex subunit 4; Mgl2, CD301b, Macrophage galactose-type lectin 2; TLR, Toll-like receptors.

Mitochondrial control of macrophage immune functions in the tumor microenvironment depends on multiple factors, ranging from metabolic byproducts that activate signalling cascades and provide substrates for enzymatic reactions, to respiratory complexes that underpin energy production and support both transcriptional and post-transcriptional programs. Limited evidence has elucidated the importance of complex I, II, III and IV for the regulation of pro-tumoral markers, IL-10 and IFN production, respectively (**Figure 2**).

**Figure 3:**
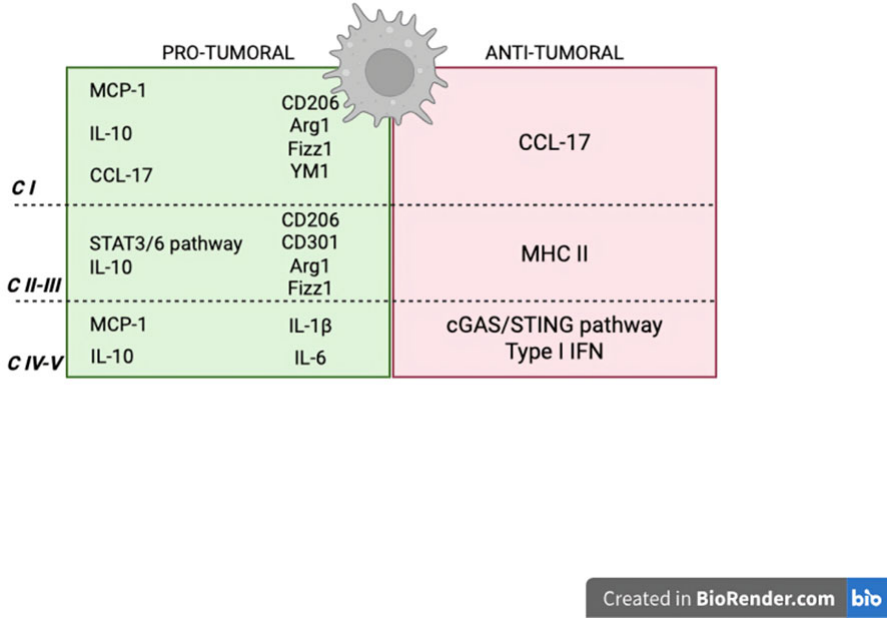
TAM pro-tumoral and anti-tumoral characteristics affected by the ETC. Complex I is involved in the modulation of the production of MCP-1 and IL-10 and of the expression of the canonical pro-tumoral markers of TAMs. It also regulates CCL-17, which has been shown to have both pro-tumoral and anti-tumoral functions. Complex II and III sustain the anti-inflammatory STAT/IL-10 pathway and promote TAMs pro-tumoral phenotype. Additionally, Complex III boosts the expression of MHC II on macrophages in the TME. Complex IV and V contribute to the release of MCP-1, IL-10, IL-6 and IL-1β by TAMs, while the impairment of Complex IV alone is responsible for the activation of the cGAS/STING pathway and subsequent IFN production. MCP-1, monocyte chemoattractant protein; IL, interleukin; TAM, tumor-associated macrophages; CCL-17, chemokine (C-C) ligand; STAT, signal transducer and activators of transcription proteins; MHC, major histocompatibility complex; cGAS/STING, cyclic GMP-AMP synthase- stimulator of interferon genes; IFN, interferon.

The findings summarized here demonstrate that the ETC is a critical regulator of TAM functionality. Remodelling of individual respiratory complexes can promote pro-tumoral phenotypes, reprogram macrophage activation states, and restrain anti-inflammatory responses in a context-dependent manner (**Figure 3**). These alterations have downstream consequences on key TAM functions, including antigen presentation, migration, phagocytosis, and the recruitment and activation of adaptive immune cells. In addition, ETC-dependent rewiring influences macrophage crosstalk with stromal components—such as pericytes, fibroblasts, and endothelial cells—and contributes to the restructuring of the tumor microenvironment through the release of cytokines, chemokines, and metabolites. Ultimately, these processes determine the balance between tumor cell engraftment and immune surveillance.

The study of the electron transport chain in TAMs and its contribution to cancer progression has slowly advanced over the past decade due to critical challenges. Firstly, the investigation of the bioenergetic machinery in mitochondria remains technically demanding, as redox cofactors are usually difficult to quantify in a reliable way and ROS rapidly interconvert in multiple reactive derivatives to increase their stability, thus hindering accurate measurements. Secondly, the complexity of the TME makes the isolation of TAMs and the analysis of their crosstalk with cancer cells and other immune cells extremely problematic, as cellular interactions evolve over time and in response to therapy-induced perturbations. Thirdly, the context-dependent and highly plastic nature of macrophage further hampers the comparison of ETC-related findings across experimental models and disease settings.

In light of these limitations in the current literature, several fundamental questions arise about the respiratory profile of macrophages when in tumoral context, given that TAMs are described as phenotypically and functionally diverse (SPP1+, TREM2+, FOLR2+, IL-1β+ macrophages) in different type and stages of cancer disease (primary tumor/metastasis; naive patients/treated patients). Does the activity of the ETC correlate with prognosis or response to therapy? What are the defining differences in mitochondrial respiration between TAMs and tissue-resident macrophages? Despite their distinct ontogeny, these macrophage populations share overlapping effector functions and can both contribute to tumor establishment or eradication in specific organs, attempting to preserve tissue homeostasis.

ETC-dependent reprogramming of TAMs or Chimeric Antigen Receptor (CAR)-M cell engineering through mitochondria functionality (as it is now being shown with cytokine-armed CAR-T cells (34)) could represent a promising therapeutic strategy, albeit with several challenges. For instance, Complex I inhibition in mice has been proven beneficial against melanoma progression but its effects are impacting both tumoral and immune cells (35). Numerous studies involving the inhibition of Complex I activity and ROS generation in cancer cells have demonstrated its therapeutic potential in primary tumor (36) and cancer metastasis (37, 38), but failed in first clinical trials due to high toxicity (34). Therefore, the specificity and side effects of ETC blockers must be systematically evaluated - a process that will likely shape future research in this area over the next decade.
